# Supplementary material for: Observation of a spontaneous anomalous Hall response in the Mn5Si3 d-wave altermagnet candidate
Source: Nat Commun. 2024 Jun 11;15:4961. doi: 10.1038/s41467-024-48493-w (PMC11167012; doi:10.1038/s41467-024-48493-w)
Supplement: Supplementary file 1 — Supplementary Information [file 41467_2024_48493_MOESM1_ESM.pdf]

Supplementary information: Observation of a spontaneous anomalous Hall response in the Mn_5Si_3 d-wave altermagnet candidate

Helena Reichlova,[1,2,*] Rafael Lopes Seeger,[3,*] Rafael González-Hernández,[4,5] Ismaila Kounta,[6] Richard Schlitz,[1] Dominik Kriegner,[1,2] Philipp Ritzinger,[1,2] Michaela Lammel,[7,8] Miina Leiviskä,[3] Anna Birk Hellenes,[5] Kamil Olejnik,[2] Vaclav Petřiček,[2] Petr Doležal,[9] Lukas Horak,[9] Eva Schmoranzerova,[10] Antonín Badura,[10] Sylvain Bertaina,[11] Andy Thomas,[1,7] Vincent Baltz,[3] Lisa Michez,[6] Jairo Sinova,[5,2] Sebastian T. B. Goennenwein,[1,12] Tomáš Jungwirth,[2,13] and Libor Šmejkal[5,2]

[1]*Institut für Festkörper- und Materialphysik and Würzburg-Dresden Cluster of Excellence ct.qmat, Technische Universität Dresden, 01062 Dresden, Germany*

[2]*Institute of Physics, Czech Academy of Sciences, Cukrovarnická 10, 162 00, Praha 6, Czech Republic*

[3]*Univ. Grenoble Alpes, CNRS, CEA, Grenoble INP, Spintec, F-38000 Grenoble, France*

[4]*Grupo de Investigación en Física Aplicada, Departamento de Física, Universidad del Norte, Barranquilla, Colombia*

[5]*Institut für Physik, Johannes Gutenberg Universität Mainz, 55128 Mainz, Germany*

[6]*Aix Marseille Univ, CNRS, CINAM, AMUTECH, Marseille, France*

[7]*Leibniz Institute for Solid State and Materials Research Dresden (IFW Dresden), Institute for Metallic Materials, 01069 Dresden, Germany*

[8]*Universität Konstanz, Fachbereich Physik, 78457 Konstanz, Germany*

[9]*Department of Condensed Matter Physics, Faculty of Mathematics and Physics, Charles University, Ke Karlovu 5, 121 16 Prague 2, Czech Republic*

[10]*Department of Chemical Physics and Optics, Faculty of Mathematics and Physics, Charles University, Ke Karlovu 5, 121 16 Prague 2, Czech Republic*

[11]*Aix Marseille Univ, CNRS IM2NP-UMR 7334 Marseille, France*

[12]*Universität Konstanz, Fachbereich Physik,78457 Konstanz, Germany*

[13]*School of Physics and Astronomy, University of Nottingham, NG7 2RD, Nottingham, United Kingdom*

* These two authors contributed equally

SUPPLEMENTARY NOTE 1: EXTENDED CHARACTERISATION DATA

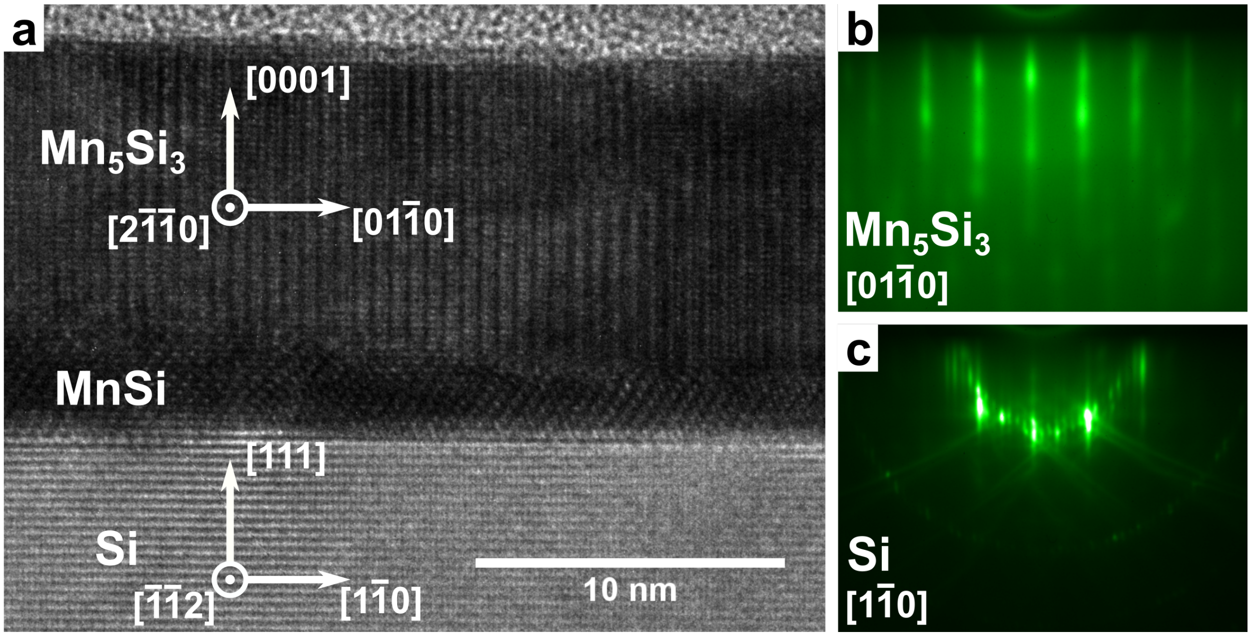

Supplementary Figure 1. Growth characterization of the epitaxial Mn_5Si_3 films. **a** High resolution transmission electron microscopy image showing a $\sim$ 12 nm thick Mn_5Si_3 film together with a thin layer of MnSi formed at the substrate interface. The crystalline orientation of the substrate and Mn_5Si_3 are labeled. Reflection high-energy electron diffraction patterns taken along **b** Mn_5Si_3 [01$\bar{1}$0] and **c**Si [1$\bar{1}$0] azimuths showing the $(\sqrt{3} \times \sqrt{3})$R30° reconstruction observed during the growth of a Mn_5Si_3 film.

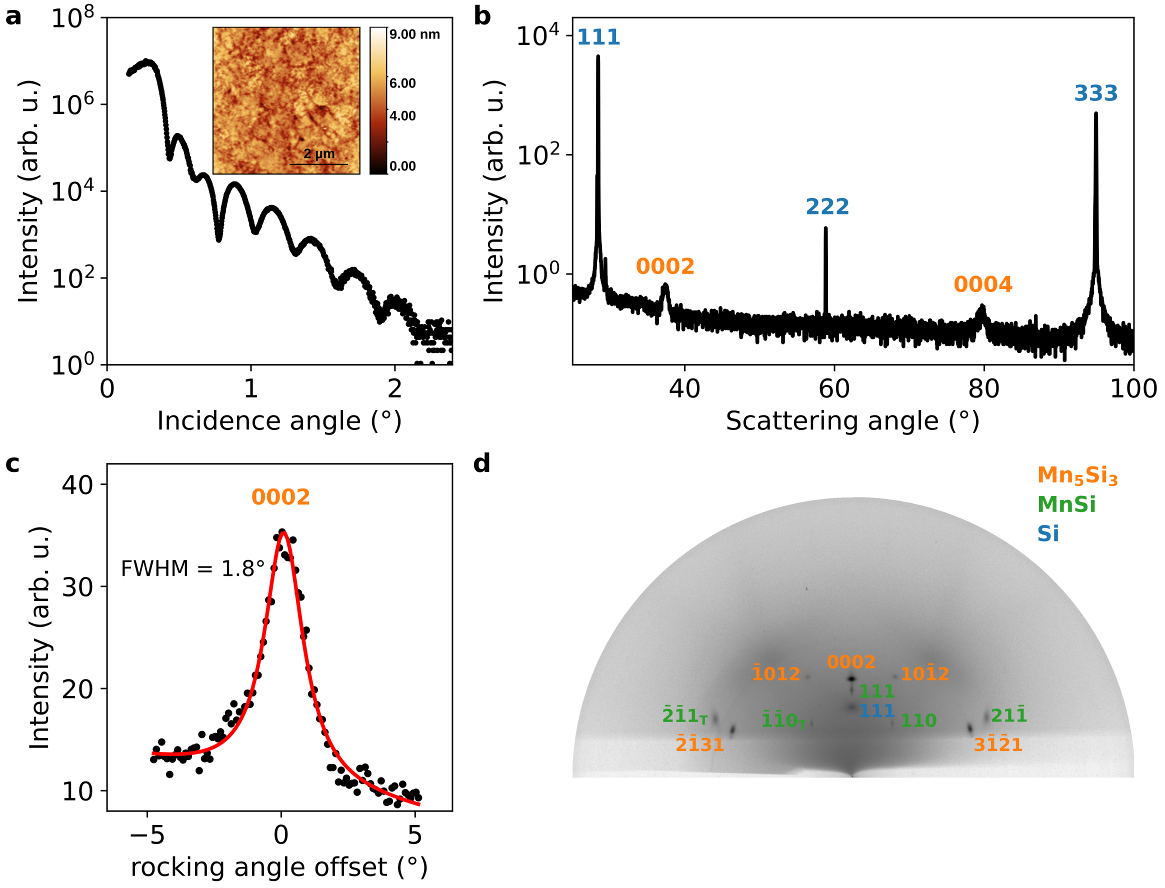

Supplementary Figure 2. Structural characterization by atomic force microscopy (AFM) and X-ray reflectivity and diffraction. **a** X-ray reflectivity data of a ~ 12 nm thick epilayer together with an AFM image shown in the inset. The experiments evidence low interface and surface roughness. In particular, the AFM image corresponds to a mean roughness $R_a = 0.7$ nm. **b** X-ray diffraction radial scan showing a diffraction signal of lattice planes parallel to the film surface. Due to the epitaxial alignment and (111) substrate surface exclusively the set of $000L$ Bragg peaks of Mn_5Si_3 are observed. **c** X-ray diffraction rocking curve of the Mn_5Si_3 0002 Bragg diffraction (points) overlaid with a Lorentzian peak fit (solid line). The mosaic broadening as determined from the FWHM is 1.8°. **d** Two-dimensional diffraction pattern obtained by acquisition of the diffraction signal while scanning the incidence angle of the X-ray beam with respect to the sample surface from 16 to 20°. Several Bragg peaks of the MnSi and Mn_5Si_3 epilayers and substrate can be identified and are labelled with their respective Miller or Miller-Bravais indices. For MnSi Bragg reflections, "T" denotes reflections from film volume with inverted/twinned stacking order [11]. The absence of Debye rings shows epitaxial growth. All diffraction data were recorded with Cu radiation.

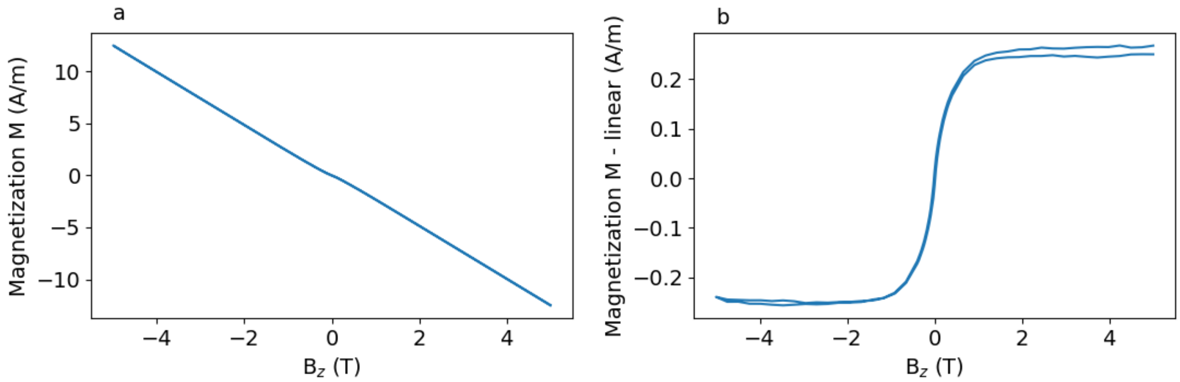

Supplementary Figure 3. Magnetometry of the Si(111) substrate. (a) As measured data at 100 K. (b) Linear background subtracted to highlight the non-linearity.

SUPPLEMENTARY NOTE 2: EXTENDED MAGNETO-TRANSPORT DATA

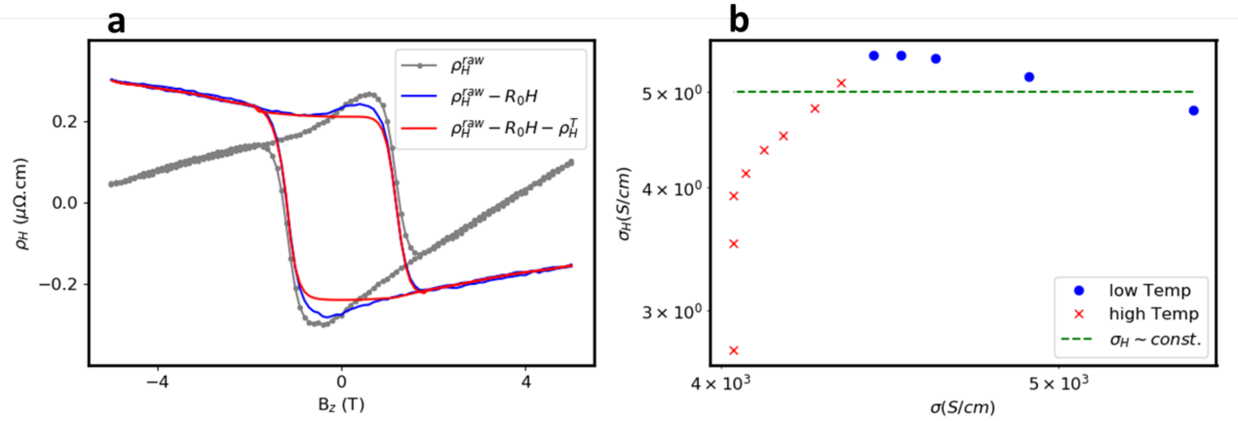

Supplementary Figure 4. **a** Extraction of spontaneous and topological Hall contributions. From the measured data, a linear background corresponding to an ordinary Hall effect was subtracted. From the remaining data the topological contribution, determined as described in the Methods section of the main text, was subtracted and the remanent spontaneous value at zero magnetic fields is attributed to the antiferromagnetic Hall effect. **b** The relationship between the transverse and longitudinal conductivity suggests the intrinsic origin of the Hall conductivity. The collinear and non-coplanar magnetic phases are distinguishable and are shown as high and low-temperature data points, respectively.

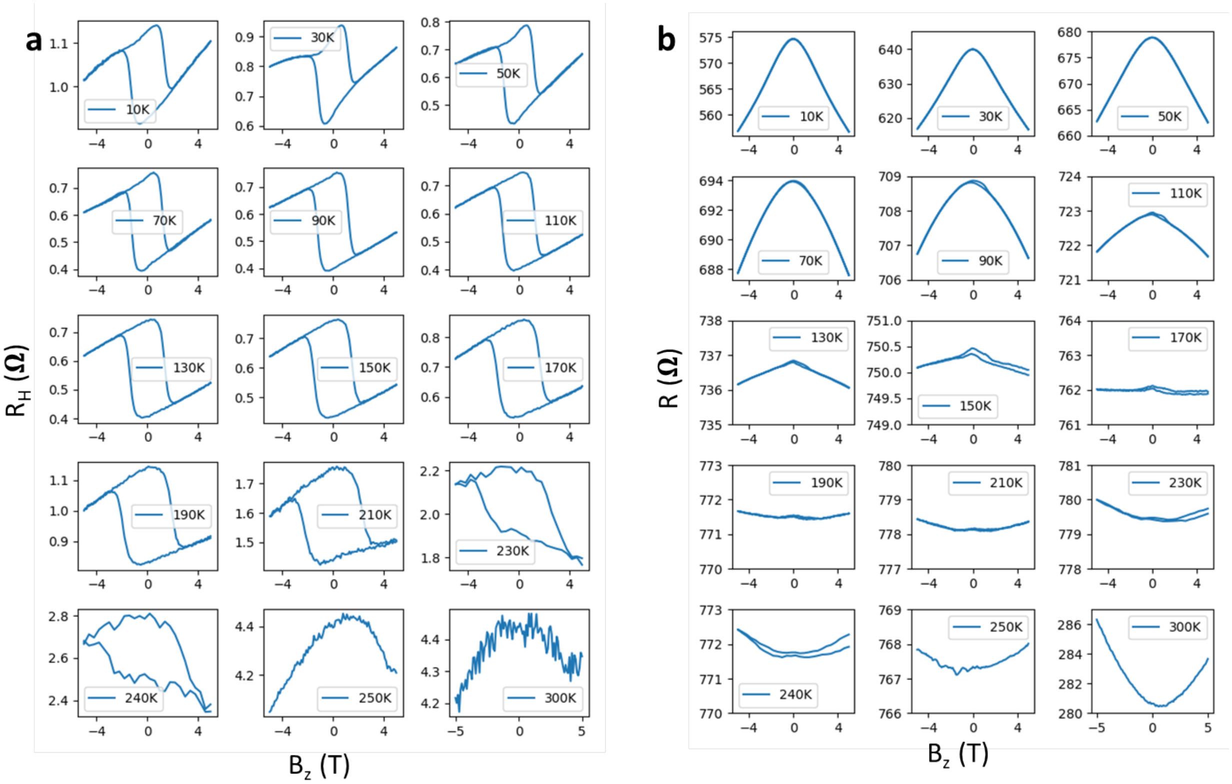

Supplementary Figure 5. Transverse (left) and longitudinal (right) magneto-resistance in a Hall bar for out-of-plane magnetic field sweeps at various temperatures. At each data point, a DC electric current of +0.1 mA and -0.1 mA was applied and the measured resistance values were averaged (so-called delta method) to compensate for possible thermo-electric contributions (voltage offsets). Data are shown prior background subtraction and anti-symmetrisation. The most prominent characteristics - clear remanence and a large coercive field in case of transverse resistance are evident for temperatures up to 240 K. Longitudinal magneto-resistance is suppressed in the collinear phase. Note the changing y-axis scale of the various sub-panels.

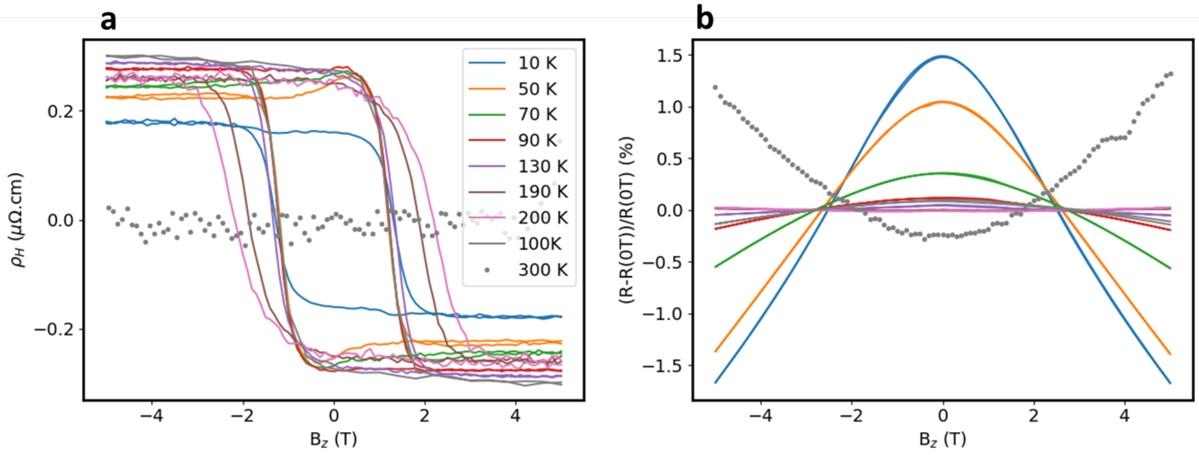

Supplementary Figure 6. Anomalous Hall resistivity measured at various temperatures (panel **a**) and simultaneously measured magneto-resistance (panel **b**) plotted in one figure for better comparison. Linear background subtracted.

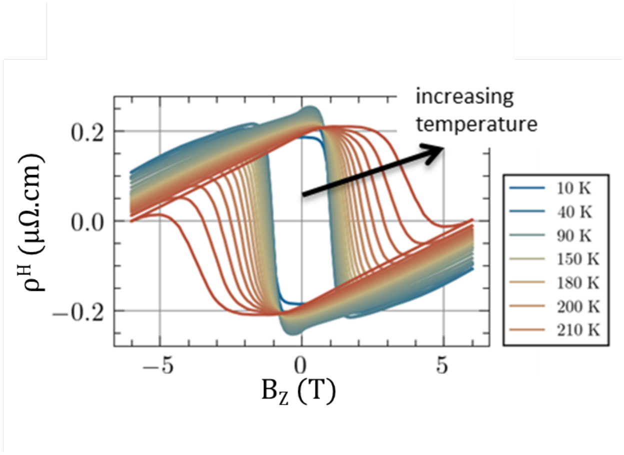

Supplementary Figure 7. Anomalous Hall effect in a Mn_5Si_3 epilayer measured at various temperatures. We observe an increase of the reorientation field (coercivity) with increasing temperature.

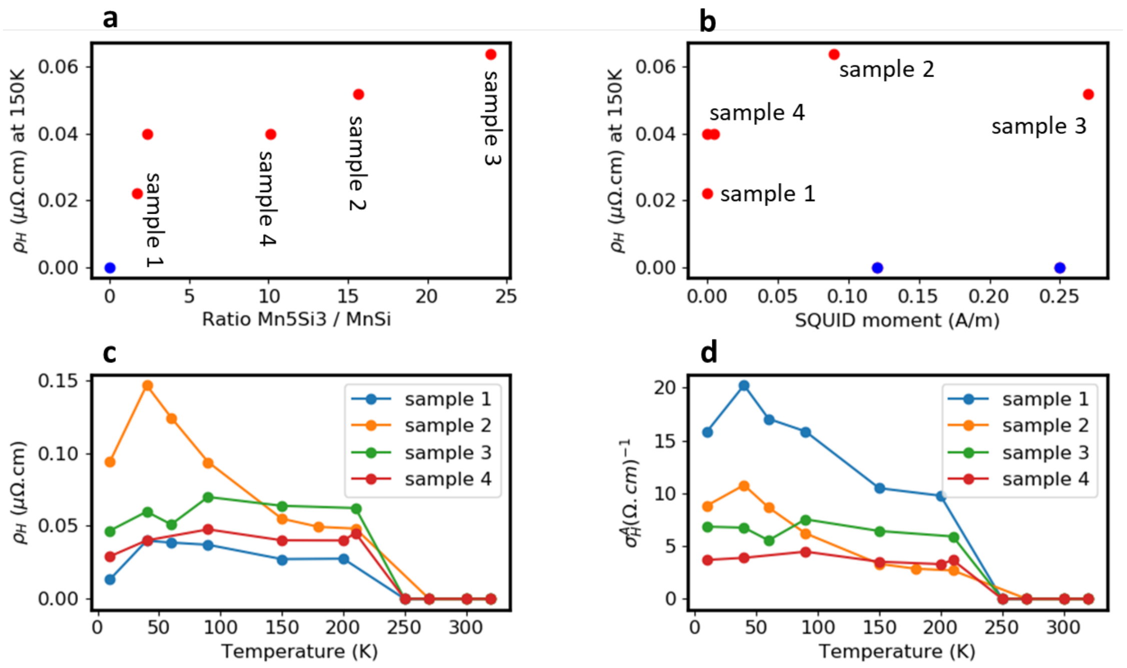

Supplementary Figure 8. Spontaneous Hall effect data for various epilayers with varying phase purity. **a** The measured Hall resistivity is correlated with the Mn_5Si_3 phase purity: Higher phase purity, indicated by a higher ratio of Mn_5Si_3 vs. MnSi phase extracted from X-ray diffraction data corresponds to higher Hall resistivity. We have used MnSi(111) peak and Mn5Si3 (0002)peak for the estimation. This excludes an impurity origin of our signal and is in general in contrast to the behavior expected for the spontaneous anomalous Hall effect in simple ferromagnets. The nominal thickness of the samples is 13 nm, 12 nm, 23 nm, 25 nm for samples 1, 2, 3 and 4, respectively. **b** In clear contrast to panel (a) the measured SQUID moment cannot be correlated to the measured anomalous Hall resistivity as shown in panel (b). **c** Temperature dependence of the Hall resistivity for various samples. While different samples with varying phase purity exhibit a variation in the Hall resistivity amplitude, the general trend, non-zero spontaneous Hall resistivity up to approximately 240 K and zero above, remains. **d** The same trend is observed also for the spontaneous Hall conductivity of this sample set. The measured value of 5-20 S/cm is in excellent agreement with theoretical calculations. We attribute the variations in the longitudinal resistivity of the studied samples to variations in the contribution to scattering from interface/surface roughness.

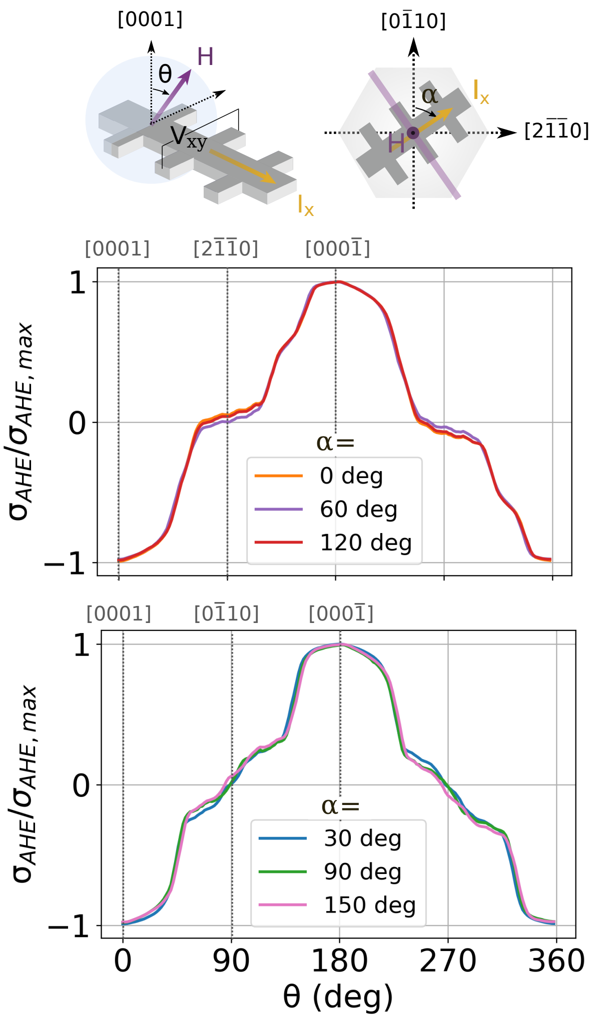

Supplementary Figure 9. Anisotropic AHE in rotating 4 T field. Top left panel: magnetic field is rotated from the normal-to-the-plane [0001]-axis ($\theta = 0$) to an in-plane direction ($\theta = 90$ deg). Top right panel: definition of the in-plane angle α. Because only four out of six Mn2 atoms are magnetic, there are three crystallographically equivalent possibilities for the distribution of these magnetic Mn2 atoms (three equivalent Mn2 "quadruplets"). This in effect can restore the hexagonal symmetry of the magnetic responses, including the AHE, as confirmed by measurements in the strong saturating magnetic field rotated in the (0$\bar{1}$10)-plane and the two other crystallographically equivalent planes (middle panel), and in the (2$\bar{1}\bar{1}$0)-plane and the two other crystallographically equivalent planes (bottom panel). The AHE traces for field rotations in crystallographically equivalent planes fall on top of each other, while there is a clear distinction between the non-equivalent planes. Note also that these AHE rotation traces show a strong anisotropy, i.e., a strong deviation from a $\cos\theta$ dependence. In contrast, the measured weak magnetization is isotropic, providing additional confirmation that the AHE is not correlated to the weak magnetization.

SUPPLEMENTARY NOTE 3: REFERENCE STUDY ON MnSi/Si(111) EPILAYER

We have grown and characterized a control thin (8 nm) epitaxial film of MnSi on Si(111). In the following we present measurements on this control sample, summarized in Figs. S10,11. We confirm the MnSi crystal structure and the quality of the film by X-Ray diffraction. We observe a ferromagnetic transition temperature below 50 K, consistent with earlier studies [1–3]. From the measured resistivity of the control MnSi thin film we conclude that less than 10 % of the current in our Si(111)/Mn_5Si_3 films is shunted by the MnSi seed layer. We detect an anomalous Hall effect in the control MnSi thin film below 50 K in an applied out-of-plane field. It shows the characteristic non-hysteretic hard-axis field-sweep dependence. This confirms that the thin MnSi seed layer in our epitaxial Si(111)/Mn_5Si_3 films cannot explain the dominant characteristics of the anomalous Hall signal, including the remanence and the 2-3 T coercivity, observed over the broad temperature range.

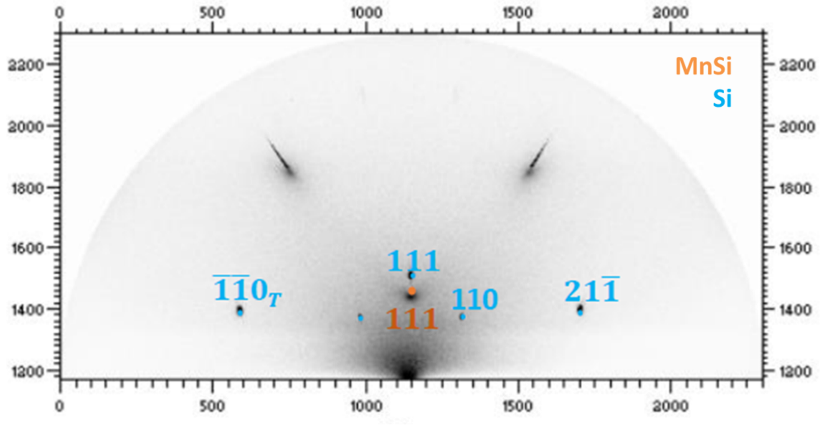

Supplementary Figure 10. X-ray diffraction radial scan of the reference sample with 8 nm thick MnSi film.

SUPPLEMENTARY NOTE 4: FIRST-PRINCIPLES CALCULATIONS OF GROUND STATE ENERGY, FERMI SURFACES, AND BERRY CURVATURE

Ground state energy and easy axis

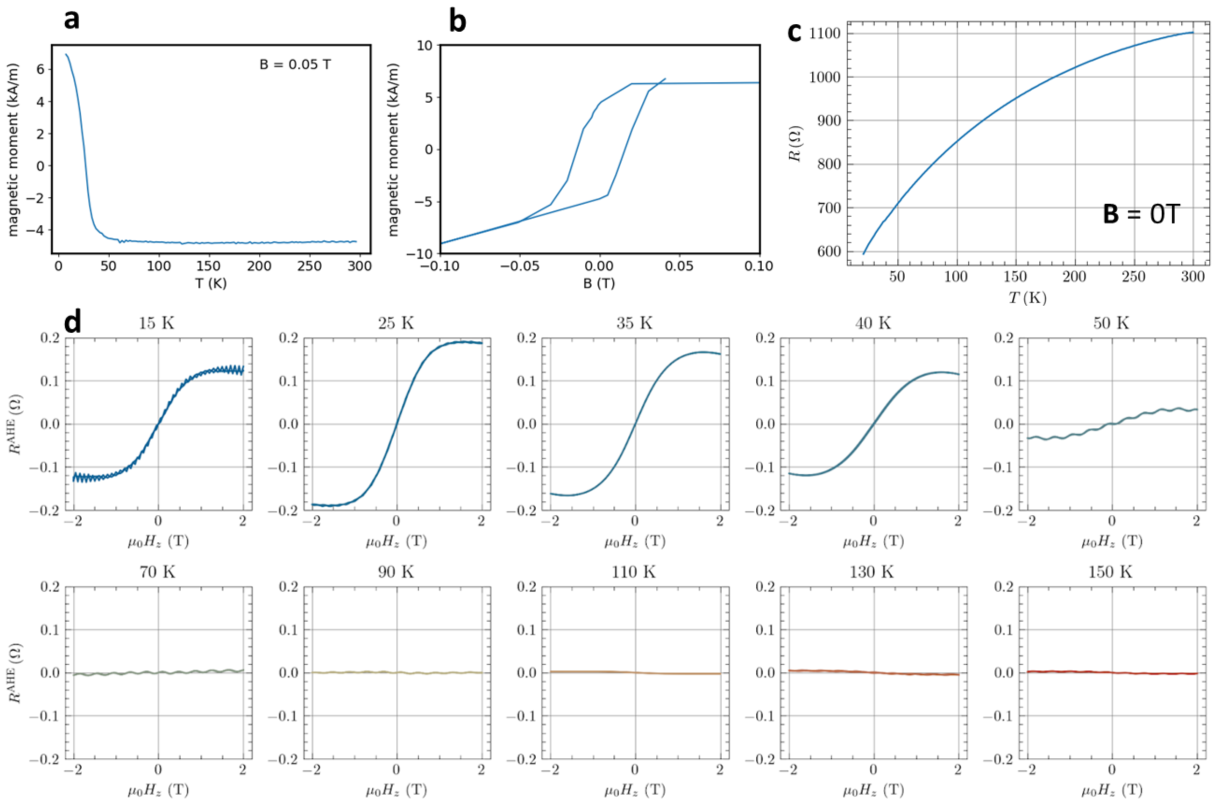

Supplementary Figure 11. Reference measurements of 8 nm MnSi film grown on Si(111) substrate. (a) Magnetometry confirms the critical temeprature of MnSi to be between 40 K and 50 K, therefore it was not enhanced significantly by growth on the Si (111) substrate. (b) Magnetization loop measured along the in-plane directions confirms the easy in-plane axis, however, the coercive field is also along the easy axis significantly smaller (50 mT) compared to Mn_5Si_3. (c) Temperature dependent longitudinal resistance does not show any transitions at temperatures above 50 T. (d) Magneto-transport data - anomalous Hall effect is detected only at low temperatures (up to 50 K), no hysteresis is visible which confirms that the measured signal in Mn_5Si_3 epilayers cannot be attributed to the MnSi spurious phase.

We summarize the ground-state energies and the sublattice and total magnetisations in the following table (Mn_{1A} and Mn_{1B} refer to the two types of the 6 Mn_1 atoms).

We observe that the d-wave altermagnetic phase exhibits the lowest energy. Furthermore, we focus on determining the magnetocrystalline energy for the different orientations of the magnetic moments with respect to the crystallographic axes. Our ground state energy calculations with spin-orbit interaction confirm the preference of a low symmetry orientation

Ordering	$\Delta E/eV$	Mn_{1A} and Mn_{1B} $/\mu_B$	Mn_2 $/\mu_B$	Magnetisation per M $/\mu_B$
**Nonmagnetic**	0.95	0.00	0.00	0.00
**Ferrimagnetic**	0.96	-0.37	0.43	-0.05
**Altermagnet**	0.00	2.54 and 0.00	<0.01	0.00

Supplementary Table I. Ground-state energies and the sublattice and total magnetisations.

of the Néel vector ([111] direction) over in-plane orientation and out-of-plane orientation by 0.9 and 0.2 meV/unit cell, respectively.

In the following table we summarize the ground-state energies for the two phases considered in the main text Fig.1a and b with the strained lattice parameters and relaxed atomic positions.

Ordering	E/eV	a/A	c/A
**Altermagnet**	-127,46	6,9500	4,7900
**Antiferromagnet**	-127,59	6,9500	4,7900

Supplementary Table II. Ground-state energies.

The resulting energy for the antiferromagnetic state is 0.13 eV per the unit cell lower than for the altermagnetic state . We point out that in reality altermagnetic state can be realized in local energy minium and that also such a small energy difference can be overturned by interfacial effects in the heterostructure. It would be interesting to account for these effects in future computational studies.

Fermi surfaces

In Fig. S12, we compare non-relativisitic Fermi surfaces with spin-orbit coupling turned off (Fig. S12a) and relativistic Fermi surfaces with spin-orbit coupling turned on (Fig. S12b). We see that the primary energy scales are governed by non-relativistic physics.

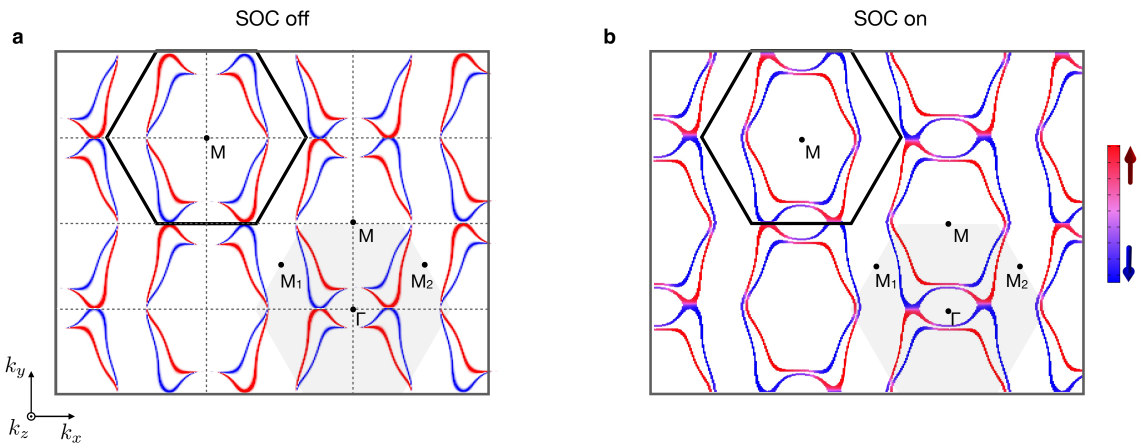

Supplementary Figure 12. Fermi surface calculations in Mn_5Si_3: **a** without spin-orbit interaction, **b** with spin-orbit interaction. The grey shaded hexagon marks Brillouin zone and highlights d-wave character around Γ point. The bold black line hexagon highlights the d-wave character around the M point.

Berry curvature maps

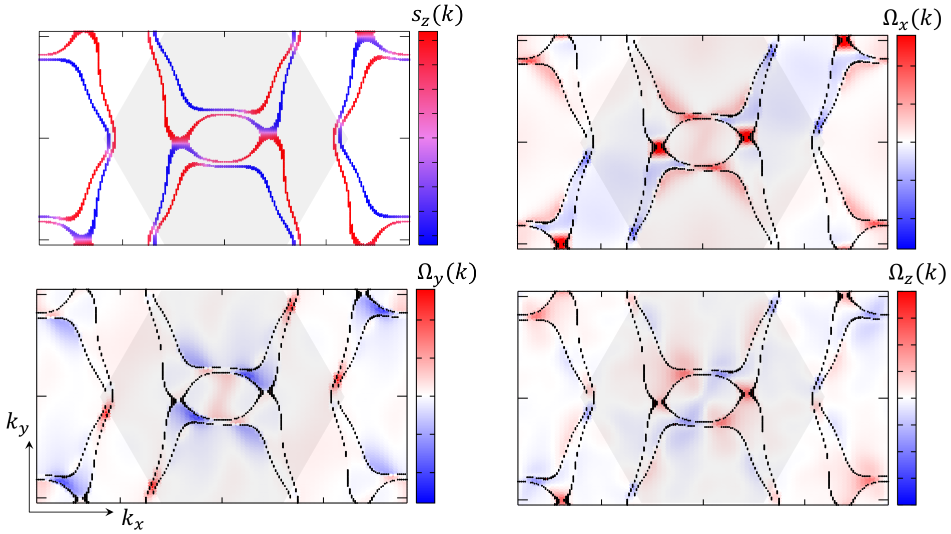

Supplementary Figure 13. Berry curvature in Mn_5Si_3: **a** Fermi surface with indicated out-of-plane spin polarization. **b-d** Momentum resolved Berry curvature map components at the $k_z = 0$ plane. The gray shaded hexagon marks Brillouin zone. Largest contributions to the Berry curvature originate from areas close to the avoided crossings generated by splitting the nonrelativistic d-wave nodal surfaces by spin-orbit coupling.

SUPPLEMENTARY NOTE 5: COMMENT ON THE SPIN STRUCTURE VISUALISATION IN MN_5SI_3 THIN FILMS

Given the fascinating properties of Mn_5Si_3 films, further efforts should be dedicated to the direct detection of magnetic order. Direct experimental detection presents significant challenges due to a variety of factors. Below, we outline potential techniques, their associated challenges, and include several promising approaches that could be utilized in the future to confirm the proposed spin structure of Mn_5Si_3 films.

REXS. Resonant X-Ray scattering was used to determine the magnetic ordering of RuO_2 thin films [12] across the Ru $\mathbf{L}_2$ edge which is around 3keV. In our sample, the Mn $\mathbf{L}_2$ edge is around 600 eV and it would require a magnetic unit cell of at least 10 Angstroms. Our Mn_5Si_3 magnetic unit cell is smaller than 7 Angstroms. On the Mn **K** edge (6 keV) the s-electrons are probed which will not yield the desired information about the magnetic ordering from the d-orbitals.

DPC-STEM. Differential-phase-contrast scanning transmission electron microscopy was used to visualise antiferromagnetic spins previously [13]. However, to achieve sufficient contrast the crystal geometry needs to be favorable. The atoms need to be very well aligned in columns with the same spin which is not the case with the orderings in Mn_5Si_3. Moreover, DPC-STEM requires the application of around 1.7 T magnetic field. The magnetic field can change the spontaneous orientation of the spins. Additionally, the DPC-STEM specimen must be prepared as a freestanding lamella. Given that the absence or relaxation of strain in our thin films might alter their magnetic properties, careful preparation of the lamella is crucial. It is essential to ensure that the films do not detach from the substrate and that the epitaxial strain remains unchanged.

Neutron scattering. The limited signal in neutron diffraction experiments, due to the small volume of thin films with a thickness of 10 nm, is a well-recognized challenge. However, this method can become highly effective for determining magnetic structures when multiple film samples are stacked together. Another challenge involves the fitting of measured spectra, which may lead to ambiguity in distinguishing between different magnetic structure fits.

X-ray Magnetic Circular Dichroism (XMCD) Altermagnetism allows for XMCD similarly as it allows for AHE. Unlike AHE which allows only to probe the out of plane component of the Hall vector, XMCD in thin films is not limited by the film geometry and it can

therefore provide vital insights into the magnetic structure of the material. Recent studies have predicted and observed XMCD signals in altermagnets [14]. Therefore, implementing XMCD experiments on Mn_5Si_3 films could yield significant findings. Combination of the linear and circular dichroism can provide further information about the spin orientation. Adding real space imaging by X-ray Vector Nanotomography [15] can bring information about spatial distribution of the magnetic order.

Spin-Resolved Angle-Resolved Photoemission Spectroscopy While Spin ARPES might not provide a definitive characterization of the spin structure, its recent successful application in demonstrating spin splitting in altermagnetic MnTe highlights its potential. Applying the same technique to Mn_5Si_3 films could be insightful. However, given the surface sensitivity of Spin ARPES, it's crucial to either cap the samples or, ideally, grow them directly at the beamline to ensure a clean sample surface. Note that, unlike MnTe, bulk samples of Mn_5Si_3 cannot be used for this purpose, as they do not exhibit altermagnetism.

In addition to directly imaging the magnetic structure, more experimental efforts are necessary to understand the emergence of the proposed magnetic phase in Mn_5Si_3. These efforts might include: (1) Investigating the role of the substrate by experimenting with alternatives, such as GaAs, which is epitaxially compatible. Eventually test the role of buffer composition to tune the lattice matching/strain. (2) Studying the influence of strain, both by applying strain to the films and potentially to the bulk material. This could reveal whether altermagnetism can be modulated or toggled by external strain. (3) Conducting a stoichiometric series analysis of $Mn_5Si_xGe_{1-x}$. Since Mn_5Ge_3 is a ferromagnetic compound, substituting Si for Ge alters the interatomic distances. It would be intriguing to determine at what point the compound becomes compensated and altermagnetic and to thoroughly investigate the role of interatomic distance in this context.

[1] Zhang, S.L.. *et al.* Engineering helimagnetism in MnSi thin films. *AIP Advance* **6**, 015217 (2016).

[2] Schroeter, D. *et al.* MnSi nanostructures obtained from epitaxially grown thin films: magnetotransport and Hall effect. *Journal of Physics: Condensed Matter* **30**, 235805 (2018).

[3] Glushkov, V. *et al.* Anomalous Hall effect in MnSi: intrinsic to extrinsic crossover. *ZhETF* **101**, 512-517 (2015).

[4] Gottschilch, M. *et al.* Study of the antiferromagnetism of Mn5Si3: an inverse magnetocaloric effect material. *J. Mater. Chem.* **22**, 15275-15284 (2012).

[5] Brown, P. J. *et al.* The low-temperature antiferromagnetic structure of Mn5Si3 revised in the light of neutron polarimetry. *J. Phys.: Condens. Matter* **4**, 10025 (1992).

[6] Litvin, D. & Opechowski, W. Spin groups. *Physica* **76**, 538–554 (1974).

[7] *Computing Solids Models, ab-initio methods and supercomputing Lecture Notes* (Forschungszentrum Julich, 2014).

[8] Strange, P. *Relativistic Quantum Mechanics* (Cambridge University Press, Cambridge, 1998).

[9] Landau, L. D. L. D., Pitaevskiĭ, L. P. L. P. & Lifshitz, E. M. *Electrodynamics of Continuous Media, Course of Theoretical Physics* (Pergamon, Oxford, 1984), vol. 8 edn.

[10] Dresselhaus, M. S., Dresselhaus, G. & Jorio, A. *Group Theory* (Springer Berlin Heidelberg, Berlin, Heidelberg, 2008).

[11] Geisler, B. *et al.* Growth mode and atomic structure of mnsi thin films on si(111). *Phys. Rev. B* **86**, 115428 (2012).

[12] Zhu, Z. H. *et al.* Anomalous Antiferromagnetism in Metallic RuO2 Determined by Resonant X-ray Scattering. *Phys. Rev. Lett.* **122**, 017202 (2019).

[13] Krizek, F. *et al.* Atomically sharp domain walls in an antiferromagnet. *arxiv* , 2012.00894 (2020).

[14] Hariki, A. *et al.* X-ray Magnetic Circular Dichroism in Altermagnetic alpha-MnTe. *arXiv:2305.03588* , (2023).

[15] Donnelly, C. *et al.* Three-dimensional magnetization structures revealed with X-ray vector nanotomography. *Nature* **547**, 328–331 (2017).
